# Colonization of *Beauveria bassiana* 08F04 in root-zone soil and its biocontrol of cereal cyst nematode (*Heterodera filipjevi*)

**DOI:** 10.1371/journal.pone.0232770

**Published:** 2020-05-05

**Authors:** Jie Zhang, Bo Fu, Qitong Lin, Ian T. Riley, Shengli Ding, Linlin Chen, Jiangkuan Cui, Lirong Yang, Honglian Li

**Affiliations:** 1 Institute of Plant Protection Research, Henan Academy of Agricultural Sciences, Zhengzhou, China; 2 College of Tobacco Science, Henan Agricultural University, Zhengzhou, China; 3 College of Plant Protection, Henan Agricultural University, Zhengzhou, China; 4 Department of Plant Production and Technologies, Faculty of Agricultural Science and Technologies, Niğde Ömer Halisdemir University, Niğde, Turkey; Fujian Agriculture and Forestry University, CHINA

## Abstract

Cereal cyst nematodes cause serious yield losses of wheat in Hunaghuai winter wheat growing region in China. *Beauveria bassiana* 08F04 isolated from the surface of cysts is a promising biological control agent for cereal cyst nematodes. As the colonization capacity is a crucial criteria to assess biocontrol effectiveness for a microbial agent candidate, we aimed to label *B*. *bassiana* 08F04 for efficient monitoring of colonization in the soil. The binary pCAM-gfp plasmid containing *sgfp* and *hph* was integrated into *B*. *bassiana* 08F04 using the *Agrobacterium tumefaciens*-mediated transformation. The transformation caused a significant change in mycelial and conidial yields, and in extracellular chitinase activity in some transformants. The cultural filtrates of some transformants also decreased acetylcholinesterase activity and the survival of *Heterodera filipjevi* second-stage juveniles relative to the wild-type strain. One transformant (G10) had a growth rate and biocontrol efficacy similar to the wild-type strain, so it was used for a pilot study of *B*. *bassiana* colonization conducted over 13 weeks. Real-time PCR results and CFU counts revealed that the population of G10 increased quickly over the first 3 weeks, then decreased slowly over the following 4 weeks before stabilizing. In addition, the application of wild-type *B*. *bassiana* 08F04 and transformant G10 significantly reduced the number of *H*. *filipjevi* females in roots by 64.4% and 60.2%, respectively. The results of this study have practical applications for ecological, biological and functional studies of *B*. *bassiana* 08F04 and for bionematicide registration.

## Introduction

Cereal cyst nematodes (CCNs) are recognized as the most important group of plant-parasitic nematodes of cereal crops and cause substantial yield losses in combination with other biotic and abiotic factors [1,2]. Of the 12 recognized CCN species, *Heterodera filipjevi* is one of the most damaging, causing yield losses that can reach 50% in individual fields [3,4]. Therefore, effective control of CCN is critical to maintaining global food security. CCN control usually requires the cultivation of resistant cultivars and/or rotations including non-hosts [5,6]. However, breeding resistance cultivars takes time and is complex due to the number of CCN species and pathotype variation [7]. Also, cereals are often the most profitable winter crop and non-host rotational crops may not be financially viable [8]. Consequently, development of biological control is needed to extend the options available for viable and long-term control of CCN.

Various microbial biological control agents (BCAs) of CCNs, such as *Purpureocillium lilacinum*, *Pochonia chlamydosporia* and *Bacillus firmus*, have been developed as commercial products [9,10]. In Europe, risk assessment of the colonization and population dynamics of BCAs in the environment is required for registration as a biopesticide (Regulation EC 414/1991) [11]. In addition, tracing the fate of BCA is crucial for predicting biological control efficiency and for developing optimal application plans [12]. It is thus meaningful to monitor the colonization of BCA in the environment. However, when using the standard microbiological method of counting colony forming units (CFU) on selective media, it is not possible to reliably distinguish a released strain from similar organisms resident in a soil microbial community [13]. Microbes resident in soil are greatly influenced by environmental conditions and their populations can change unpredictably [14]. So, when evaluating BCAs, it is important to determine the influence of resident genotypes and to use a method that is able to distinguish them from the exogenous strain. Consequently, real-time PCR is becoming the most commonly used method for colonization studies due to their high levels of accuracy and reproducibility.

Rhizosphere competence and the different factors affecting the establishment of inoculants can be efficiently studied under well-defined greenhouse conditions using strains genetically modified for monitoring purposes, provided containment requirements are addressed [15]. With the development of molecular biology, the exogenous gene maker technique has become a valuable tool for the study of environmental microorganism, especially with the extensive use of green fluorescent protein (GFP) and antibiotic resistance genes. *Agrobacterium tumefaciens*-mediated transformation (ATMT) serves as a useful method for foreign gene insertion into filamentous fungi through non-homologous recombination and T-DNA random insertion [16,17]. The phosphinothricin acetyltransferase gene (*bar*) has been transformed into *B*. *bassiana* by ATMT and used as a selectable marker [18]. An enhanced green fluorescent protein has also been successfully expressed in *B*. *bassiana* by ATMT and 60% of transformants have been verified to be inserted by the T-DNA fragment [19]. At present, the colonization of some BCAs have been studied by their exogenous gene-marked strain. For example, Escudero and Lopez-Llorca [20] used real-time PCR and CFU to analyze the behavior of *P*. *chlamydosporia* in tomato roots and root-knot nematode *Meloidogyne javanica*, and their results showed that there was no difference in the virulence/root colonization of wild type strain and its stable GFP transformant. In addition, the colonization of *Vitis* spp. wood by the pathogenic fungi *Phaeomoniella chlamydospora* was studied using a GFP transformant and the relative copy number of *sgfp* was estimated by real-time PCR [21]. However, there have been no published studies that have used both real-time PCR and CFU counts to monitor exogenous gene-marked *B*. *bassiana* in soil.

*Beauveria bassiana* 08F04, isolated from the surfaces of cysts [22], is a promising BCA for CCNs. Since the colonization capacities of strain is crucial for biocontrol effectiveness, we aimed to label *B*. *bassiana* 08F04 for efficient monitoring of colonization in the soil. This study has provided useful tools for its application in ecological, biological and functional studies and for bionematicide registration.

## Materials and methods

### Strains and plasmid

*Beauveria bassiana* 08F04 was isolated from the surface of cysts and deposited in the China General Microbiological Culture Collection Center (CGMCC no. 8656). *Agrobacterium tumefaciens* hypervirulent strain AGL-1 was used for ATMT. The pCAM-gfp plasmid [23] containing a T-DNA with a *sgfp* gene driven by the tox-A promoter and a hygromycin-B-phosphotransferase gene (*hph*) cassette driven by the trpC promoter from *Aspergillus nidulans* were preserved in our laboratory. The pCAM-gfp plasmid was introduced into AGL-1 as described by Bowyer [24].

### *Agrobacterium tumefaciens*-mediated transformation of *Beauveria bassiana* 08F04

#### Resistance to hygromycin B

To monitor resistance, 08F04 conidial suspensions were prepared by adding 5 mL sterile water to a 14-day-old culture grown in potato dextrose agar (PDA) and by scraping the surface gently to release the newly formed conidia. After being filtered through two layers of sterile gauze, the conidia were suspended in sterile water to a final concentration of 10^6^ conidia/mL. Then 100 μL of conidial suspension was inoculated on different PDA plates with different concentrations of hygromycin B (0, 50, 100, 200, 300, 400 and 500 μg/mL) and incubated at 25°C for 7 days.

#### *Agrobacterium tumefaciens-*mediated transformation

*Agrobacterium tumefaciens* strain AGL-1 containing the pCAM-gfp plasmid was grown on a yeast extract broth (YEB) containing 50 μg/mL kanamycin and 30 μg/mL rifampin at 28°C for 36 h. The cells were pelleted at 8,000 rpm for 2 min and rinsed twice with induction medium (IM) [25]. The pellet was finally diluted in IM to OD_600_ of 0.15. The culture was then incubated at 28°C with shaking at 200 rpm for 5–6 h until the OD600 reached 0.5–0.6. The 08F04 conidia were rinsed twice with IM and then resuspended in IM to a final concentration of 10^6^ conidia/mL. About 50 μL of 08F04 conidial suspension was mixed with 50 μL of IM-suspended AGL-1 culture. Then the mixture was spread on IM plates supplemented with 0.5 mM sucrose and nylon membranes, and co-cultivated at 23°C for 48 h. The membrane was then transferred to a selection PDA plate containing 500 μg/mL hygromycin B and 200 μg/mL cefotaxime. The plates were incubated at 25°C for 5 days. The colonies obtained were then transferred to PDA plates containing 500 μg/mL hygromycin B and incubated until conidiogenesis. To obtain monoconidial isolations, conidia from individual colonies were suspended in sterile water and added to PDA containing 500 μg/mL hygromycin B, and a single germinating conidia from each colony was selected and transferred to a PDA plate supplemented with 500 μg/mL hygromycin B. To analyze the stability of the transformants, 100 putative clones were cultured on nonselective PDA plate for 5 generations after which the clones were transferred to PDA supplemented with 500 μg/mL hygromycin B.

#### Screening and verification of transformants

To screen the transformants for growth and sporulation similar to those of wild-type *B*. *bassiana* 08F04, the mycelial and conidial yields of nine transformants were determined. The mycelium yield was determined as follows: 50 μL *Beauveria* conidial suspension (10^8^ conidia/mL) was transferred to a 100-mL tube containing 30 mL PD broth and was cultured at 25°C with shaking at 160 rpm for 5 days. Then, the dry weight of the pellet was determined after being spun at 12,000 rpm for 10 min and then dried at 50°C for 12 h. The conidial yield of the strains was determined by spreading 100 μL conidial suspension on PDA medium and incubation at 25°C for 7 days. Conidial suspensions from the newly formed colonies were then obtained using the method described above. The conidial yield was determined with a hemacytometer. The experiment was repeated twice with three replicates. The four transformants screened, as described above, were used for the following verification. To confirm the expression of *sgfp* in transformants, the hyphae of wild-type strain 08F04 and screened transformants were observed with a bright field and epifluorescence microcopy using a Nikon Eclipse Ti-E with an excitation wavelength of 465/495 nm and an emission wavelength of 513/556 nm). To detect the *sgfp* and *hph* genes in putative transformants, PCR analysis was performed. Genomic DNA of the wild-type and four transformed strains was extracted by the CTAB method. Primer pairs sGFP-F/R (sGFP-F: 5'-ATGG TGAGCAAGGGCGAGG-3', sGFP-R: 5'-TTCTGCTGGTAGTGGTCGGC-3') [26] and hph-F/R (hph-F: 5'-ATGCCTGAACTCACCGCGAC-3', hph-R: 5'-CTATTCCTTTGC CCTCGGAC-3') [27] were used to confirm the incorporation of *sgfp* and *hph* genes, respectively. The PCR amplification protocol involved an initial denaturing cycle of 10 min at 94°C, followed by 30 cycles of 94°C for 1 min, 55°C for 45 s and 72°C for 1 min.

Southern blot analyses were employed to assay the copy number of T-DNA with DIG-High Prime DNA Labeling and Detection Starter Kit (Roche Diagnostics, Mannheim, Germany). After being digested by restriction endonuclease *Xho*I, the genomic DNA was divided by agarose electrophoresis and the target band was transferred to nylon membranes. In addition, the *sgfp* gene was amplified from the pCAM-gfp plasmid and the 510-bp fragment obtained was labeled as a probe. Prehybridization, hybridization and chemiluminescent detection were applied to the blots according to the manufacturer's protocol.

### Biocontrol capacities of *Beauveria bassiana* 08F04 and the transformants

Soil infested with *H*. *filipjevi* was collected from a site in Xuchang [28] and cysts were extracted from soil by modified Fenwick flotation [29]. Second-stage juveniles (J2) were prepared according to the method described previously [30] and stored at 4°C for later use. One mL of conidial suspension (10^9^ conidia/mL) of *B*. *bassiana* 08F04 and four transformants were inoculated into 50 mL of Sabouraud dextrose medium with yeast extract [31] and grown at 25°C with constant shaking at 180 rpm for 7 days. Then, the cultures of each strain were centrifuged at 10,000 rpm for 10 min. After filtering the supernatant through a 0.45-μm membrane, the filtrates were collected and used for an assay of extracellular proteases and chitinase activity and of the effect on acetylcholinesterase (AChE) activity and survival of *H*. *filipjevi* J2. For each experimental replicate, each enzyme assay was repeated three times and *H*. *filipjevi* J2 survival assay was performed with six replicates. The entire experiment was repeated three times.

#### Extracellular protease

Total protease activity levels of each strain were measured with the Folin phenol reagent following Lowry et al. [32] using casein as a substrate. Cultural filtrates of each strain (1 mL) were mixed with 1 mL 2% (w/v) casein. The mixture was incubated at 40°C for 10 min. Then 2 mL of trichloroacetic acid (0.4 M) was added to the mixture and the assay was incubated until it had fully precipitated. The mixture was then centrifuged at 10,000 rpm for 10 min, and the supernatant was added to Folin reagent. The kinetic assay was performed using a spectrophotometer (Shimadzu Co., Tokyo, Japan) at 680 nm. One unit of extracellular protease activity was defined as the enzyme volume capable of releasing 1 μg casein/mL/min under the assay conditions.

#### Extracellular chitinase

Chitinase activity was measured by determining the release of N-acetyl-β-D-glucosaminidase (NAG) from colloidal chitin with the DNS (dinitrosalicylic acid) reagent [33]. One mL of cultural filtrate was added to 1 mL 1% (w/v) colloidal chitin and incubated at 50°C for 1 h. Then, 3 mL DNS was added to the reaction solution. Finally, the kinetic assay was performed in a spectrophotometer at 540 nm. One unit of extracellular chitinase activity was defined as the enzyme volume needed to release 1 μg NAG/mL/h.

#### *Heterodera filipjevi* J2 acetylcholinesterase

To assess the effect of biocontrol strains on *H*. *filipjevi* J2 AChE activity, 200 μL of culture filtrate and 150–200 *H*. *filipjevi* J2 was added to a microcentrifuge tube and incubated at 25°C for 48 h. *H*. *filipjevi* J2 were collected by centrifugation at 6,000 rpm for 10 min and then were mixed with 1 mL of 0.1 M phosphate buffer (pH 7.5) supplemented with 0.1% (w/v) Triton X-100. The mixture was homogenized in an ice bath. The crude enzyme was then extracted by centrifugation (10 min at 10,000 rpm). Then, 1 mL crude enzyme was added to 1 mL of 0.1 M phosphate buffer (pH 7.5), 2 mL of 0.5 mM acetylthiocholine iodide and 2 mL of 0.05 mM dithio-bis-nitrobenzoic acid. Total AChE activity was assayed immediately in the spectrophotometer at 412 nm [34]. One unit of acetylcholinesterase activity was defined as the amount of enzyme that released 1 μg 5-thio-2-nitrobenzoic acid. The crude enzyme extracted from *H*. *filipjevi* J2 treated with sterile PDB medium was used as a control.

#### *Heterodera filipjevi* J2 survival

To measure the effect of *B*. *bassiana* 08F04 and the transformants on *H*. *filipjevi* J2 survival, 100 μL of cultural filtrates and 80–100 J2 were added to a microcentrifuge tube. After the mixture were incubated at 15°C for 24 h, the viability of *H*. *filipjevi* J2 were assessed. The J2 with no detectable movement in 4% (w/v) sodium hydroxide solution within 3 min were deemed not to have survived [35]. PDB medium was used in the assay as a control.

### Greenhouse experiments

Soil infested with *H*. *filipjevi* and wheat cultivar (Wenmai 19) were prepared as described previously [36]. Seeds were germinated for 60 h at 25°C after being surface-sterilized using 1% (w/v) NaClO. One germinated seed was transplanted to a plastic pot (7 × 15 cm) containing 200 g field soil and inoculated with 5 mL transformant G10 conidial suspension (10^9^ conidia/mL) by root-irrigation. Wheat inoculated with equal parts wild-type *B*. *bassiana* 08F04 conidial suspension and sterile water were used as a control. All of samples were placed in a greenhouse at 18–20°C. The root-zone soil (0.5–1 g per plant), i.e., soil adhering to the entire root system, was collected at 2-week intervals (1 to 13 weeks) and 15 plants were sampled once in each treatment. The soil from three plants was combined into one sample and a total of five samples was collected for each treatment at each sampling time. Each sample was then subdivided into two subsamples. One was kept at 4°C for CFU measurement, and the other was frozen at -20°C for the subsequent real-time PCR assay.

In addition, the biocontrol effect of transformant G10 and *B*. *bassiana* 08F04 against *H*. *filipjevi* was evaluated after 13 weeks according to the method described before [36]. After the number of CCN females in the roots was determined, 100 females from each treatment was collected and surface sterilised in 1% sodium hypochlorite for 2 min. 20 females were spread on a PDA plate supplemented with hygromycin B (500 μg/mL), streptomycin (100 μg/mL) and kanamycin (50 μg/mL) in five replicates. After incubation at 25°C for 7 days, the surface of CCN females forming *B*. *bassiana* colonies were considered to be parasitized and the parasitism rate was calculated. The entire experiment was repeated three times.

#### Real-time PCR development

Total DNA was extracted from 500 mg soil for each sample using Thermo Scientific KingFisher Flex (Thermo Fisher Scientific, Wilmington, DE, USA) according to the manufacturer's protocol of Mag-Bind^®^ Soil DNA Kit (Omega Bio-Tek, Doraville, GA, USA). The final volume of each DNA extract was measured at 50 μL and stored in tubes at -20°C until used. DNA was quantified by spectrophotometry (NanoDrop Technologies, Wilmington, DE, USA).

Forward primer sgfp1-F (5’-CCACATGAAGCAGCACGAC-3’) and reverse primer sgfp1-R (5’-TCGATGCGGTTCACCAG-3’) was designed by Primer Express 3.0 software (PE Applied Biosystems, California, USA) according to the *sgfp* gene sequence, amplifying a 144 bp fragment. The real-time quantitative PCR assay was conducted in Mastercycler ep realplex (Eppendorf, AG, Hamburg, Germany) with a 96-well optical reaction plate. The PCR mixture included 100 nM of each primer, 10 μL of SYBR Premix Ex Taq (TaKaRa Biotech., Dalian, China) and 0.5 μL extracted template (about 5–15 ng DNA was extracted from each 500-mg soil sample) in a final volume of 20 μL. The optimized thermal cycling was performed under the following conditions: 2 min at 50°C and 10 min at 95°C, followed by 40 cycles of 15 s at 95°C and 1 min at 60°C with a final melting curve (60–95°C, 1.75°C/s). To verify the specificities of the real-time PCR, reactions with templates generated from soil absence of the transformant or with no templates were used as a negative control and those with the pCAM-gfp plasmid were used as a positive control. All assays were repeated three times.

To assess the sensitivity of the real-time PCR method, the pCAM-gfp plasmid was serially diluted (10-fold) with the final concentrations ranging from 9.2 to 9.2 × 10^−7^ ng/μL. The diluted plasmids were assayed by real-time PCR for three replicates. After copy number of the *sgfp* gene for each concentration of plasmids were calculated [37], a standard curve was generated by plotting cycle threshold (Ct) values against the log-transformed values of the copy numbers. The amplification efficiency of the real-time PCR procedure was obtained according to the equation: E = 10^−1/slope^-1, the slope of which represent the slope of standard curve.

#### Colonization in wheat root-zone soil by transformant G10

Real-time PCR and CFU counts were used to determine the number of *sgfp* gene copies and propagules in the soil at different time points. PCR procedures and analyses were performed as described above. Ct values of the *sgfp* gene in each sample were assayed, and copy numbers were calculated according to the standard curve shown above.

For CFU counts, 0.5 g of soil were suspended in 5 ml of sterilized water containing 0.01% (w/v) Tween 80. After shaking for 5 min and resting for 1 min, the soil suspensions were diluted in a 10-fold series. Then 100 μL of each dilution were spread on PDA plates supplemented with hygromycin B (500 μg/mL), streptomycin (100 μg/mL) and kanamycin (50 μg/mL) in three replicates. After incubation at 25°C for 5 days, the colonies on each plate were counted.

### Statistical analysis

All analyses were performed in R version 3.1.3 (http://www.r-project.org/) with core [38] and ggplot2 packages was installed to plot statistical graph [39]. Statistical comparisons were performed using one-way analysis of variance (ANOVA) and any significant differences were further tested using least significance difference (LSD) at *p* = 0.05. Repeatability/precision was calculated as agreement according to standard errors of measurement. To achieve the best linear fit, data generated from copy number and CFU counts were natural log transformed to normalize the distribution. Regression analysis and the Bland-Altman test [40,41] were employed to analyze the correlation between the two methods.

## Results

### *Agrobacterium tumefaciens*-mediated the transformation of *Beauveria bassiana* 08F04

Before completing the transformation, the sensitivity of the wild-type strain to hygromycin B was determined. The results show that 500 μg/mL hygromycin was able to fully inhibit *B*. *bassiana* 08F04 growth on PDA. Therefore, this concentration was used to screen the resistant colonies after the transformation. The co-cultivation of 08F04 with *A*. *tumefaciens* AGL-1 containing the pCAM-gfp plasmid resulted in the formation of positive colonies on the selective medium, whereas no colonies appeared when 08F04 was incubated with *A*. *tumefaciens* without plasmids. About 120–140 transformants were obtained from 10^6^ conidia. In addition, about 90 clones were mitotically stable after 100 putative transformants grown on non-selective PDA plate for five generations, so the percentage of mitotically stable transformants reached 90% under this transformation system.

### Screening and validation of transformants

Following the transformation, nine transformants were screened for mycelial and conidial yields (S1 Table). The mycelium yields of transformants G10, G37, G67, G85 and G94 were not significantly different from the wild-type strain *B*. *bassiana* 08F04. Furthermore, these transformants except G67 had similar conidial yields as those of the wild-type *B*. *bassiana* 08F04. Therefore, transformants G10, G37, G85 and G94 were validated and assessed for biocontrol activity.

**Fig 1:**
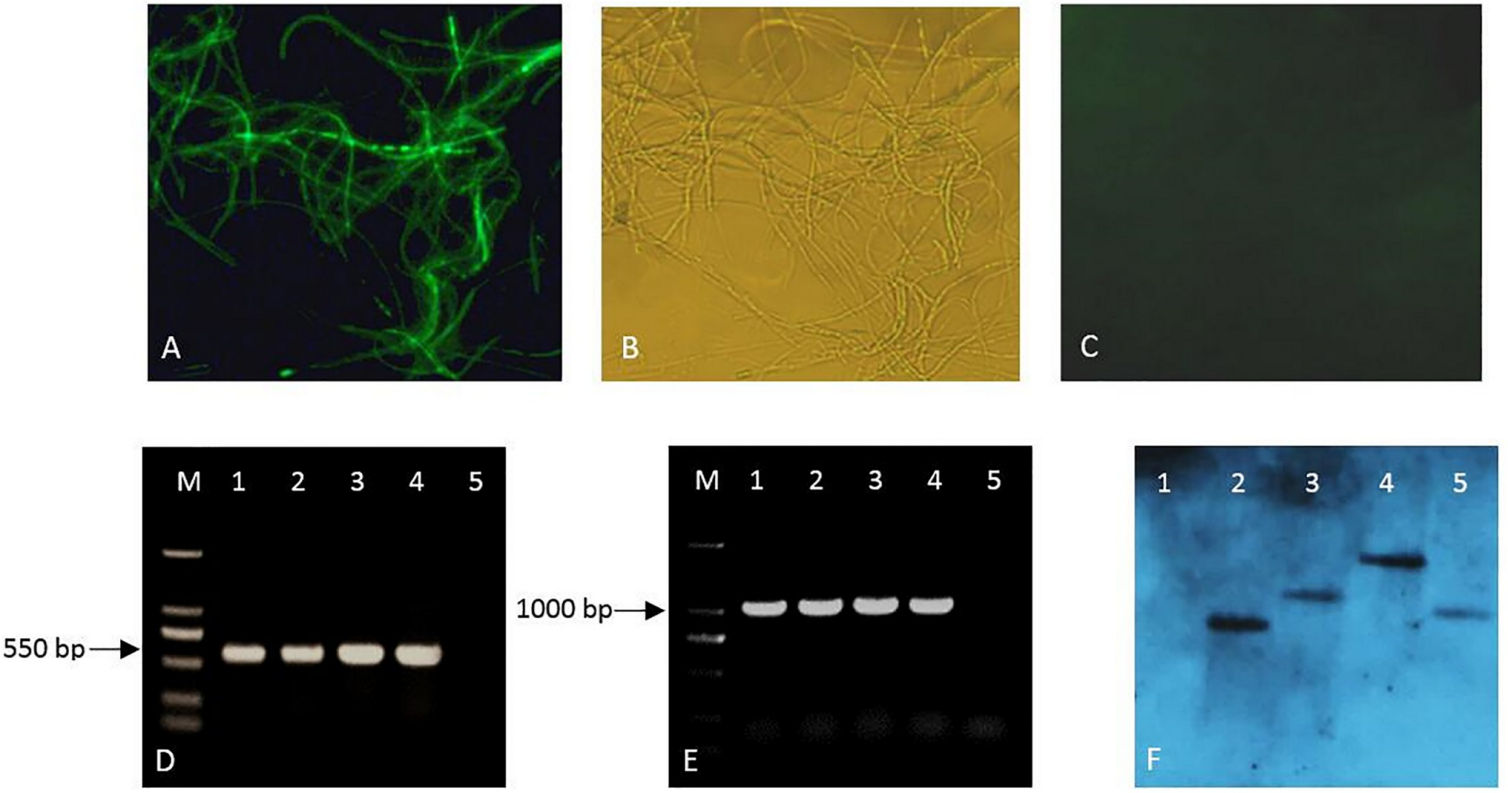
Validation of *Beauveria bassiana* 08F04 transformed with the pCAM-gfp plasmid. (A) Fluorescence microscopic observation of the transformants revealed by epifluorescence microscopy; (B) Mycelium of transformants imaged in the same microscopic field by a bright field; (C) Mycelium of wild-type *B*. *bassiana* 08F04 revealed by epifluorescence microscopy; (D) PCR products of the *sgfp* gene of the genomic DNA of the transformants (lanes 1–4: transformants G10, G37, G85 and G94) and of wild-type *B*. *bassiana* 08F04 (lane 5); (E) PCR products of the *hph* gene of the transformants (lanes 1–4: transformants G10, G37, G85 and G94) and of wild-type *B*. *bassiana* 08F04 (lane 5); (F) Southern blot analysis with a DIG-labeled *sgfp* gene probe of wild-type 08F04 (lane 1) and of the transformants (lanes 2–5: transformants G10, G37, G85 and G94).

To verify the expression of green fluorescence, mycelia were observed under fluorescence microscopy. The mycelia of the four transformants had bright green fluorescence (Fig 1A), but no autofluorescence was observed in the transformants in the same microscopic field by a bright field (Fig 1B) and wild-type *B*. *bassiana* 08F04 under fluorescence microscopy (Fig 1C). PCR analysis confirmed the presence of transgene in the four transformants. The expected sizes (550 and 1000 bp) were obtained from all of the tested transformants using sgfp-F/R (Fig 1D) and hph-F/R (Fig 1E) primers. A Southern blot analysis (Fig 1F) revealed that the four transformants integrated a single copy, and the presence of different band sizes indicated that the T-DNA gene was inserted into the genome at random.

### Biocontrol capacities of the transformants and of wild-type *Beauveria bassiana* 08F04

The biocontrol capacities of the four transformants and *B*. *bassiana* 08F04 were determined in terms of extracellular protease and chitinase activity, and acetylcholinesterase activity and survival of *H*. *filipjevi* J2 *in vitro*. The four transformants had similar patterns of protease activity as those of wild-type *B*. *bassiana* 08F04 (*F*_*4*, *10*_ = 2.8, *P* = 0.086, S2 Table). Furthermore, the chitinase activity of most transformants was not significantly different from that of *B*. *bassiana* 08F04, except in the case of G37 for which chitinase activity decreased significantly (*F*_*4*, *10*_ = 7.1, *P* = 0.006). Effects of cultural filtrates of the transformants and of *B*. *bassiana* 08F04 on acetylcholinesterase activity and juvenile survival of *H*. *filipjevi* J2 *in vitro* are shown in Table 1. The results show that cultural filtrates of transformants G10, G37 and G85, and of *B*. *bassiana* 08F04 significantly decreased the acetylcholinesterase activity in *H*. *filipjevi* J2 compared to the control, while no significant difference was observed as a result of treatment with cultural filtrates of transformant G94 (*F*_*5*, *12*_ = 8.3, *P* = 0.001). Regarding *H*. *filipjevi* J2 survival (*F*_*5*, *12*_ = 87.5, *P* = 0.0001), cultural filtrates of transformants G10, G37 and G85, and of *B*. *bassiana* 08F04, significantly decreased J2 survival, but transformant G94 did not significantly influence J2 survival relative to the control. Also, *H*. *filipjevi* J2 survival with G37 and G94 was significantly different from survival with *B*. *bassiana* 08F04. Based on the above results, transformant G10 was selected for the colonization study of *B*. *bassiana*, as the growth and biocontrol capacities of this transformant were similar to those of wild-type *B*. *bassiana* 08F04.

**Table 1:** Effect of cultural filtrates of the transformants and wild-type *Beauveria bassiana* 08F04 on acetylcholinesterase activity and survival of *Heterodera filipjevi* second-stage juveniles (J2) *in vitro*.

Strain	Acetylcholinesterase activity (U/mL)^a^	J2 survival (%)^b^
G10	2.17 ± 0.12 b	10.7 ± 0.88 d
G37	2.3 ± 0.15 b	26.3 ± 4.1 c
G85	2.23 ± 0.07 b	14.0 ±2.1 d
G94	2.76 ± 0.12 a	44.3 ± 4.7 b
08F04	2.25 ± 0.03 b	11.7 ± 0.88 d
Control	2.9 ± 0.12 a	79.3 ± 2.3 b

^a^ Data are mean ± standard error for three replicates; Values followed by the same letter within a column are not significantly different according to ANOVA and LSD test conducted at *P* = 0.05; *F*_*5*, *12*_ = 8.3, *P* = 0.001.

^b^ *F*_*5*, *12*_ = 87.5, *P* = 0.0001.

### Development of the real-time PCR

The results indicate that sgfp1-F and sgfp1-R primers specifically detected the *sgfp* gene in the real-time PCR. To assess the sensitivity of the real-time PCR procedure, serial diluted pCAM-gfp plasmids were assayed. The result showed that the assay detected as few as 7.9 × 10^1^ copies/PCR with an average Ct value of 33.4. In addition, a strong degree of linearity (y = 39.9–3.08x, *P* < 0.001, df = 6) was achieved between 7.9 × 10^1^ and 7.9 × 10^8^ copies/PCR with the correlation coefficient (R^2^) reaching 0.994 and with the amplification efficiency level reaching 111% (S1 Fig).

### Colonization and population dynamics of G10 transformant in the greenhouse

**Fig 2:**
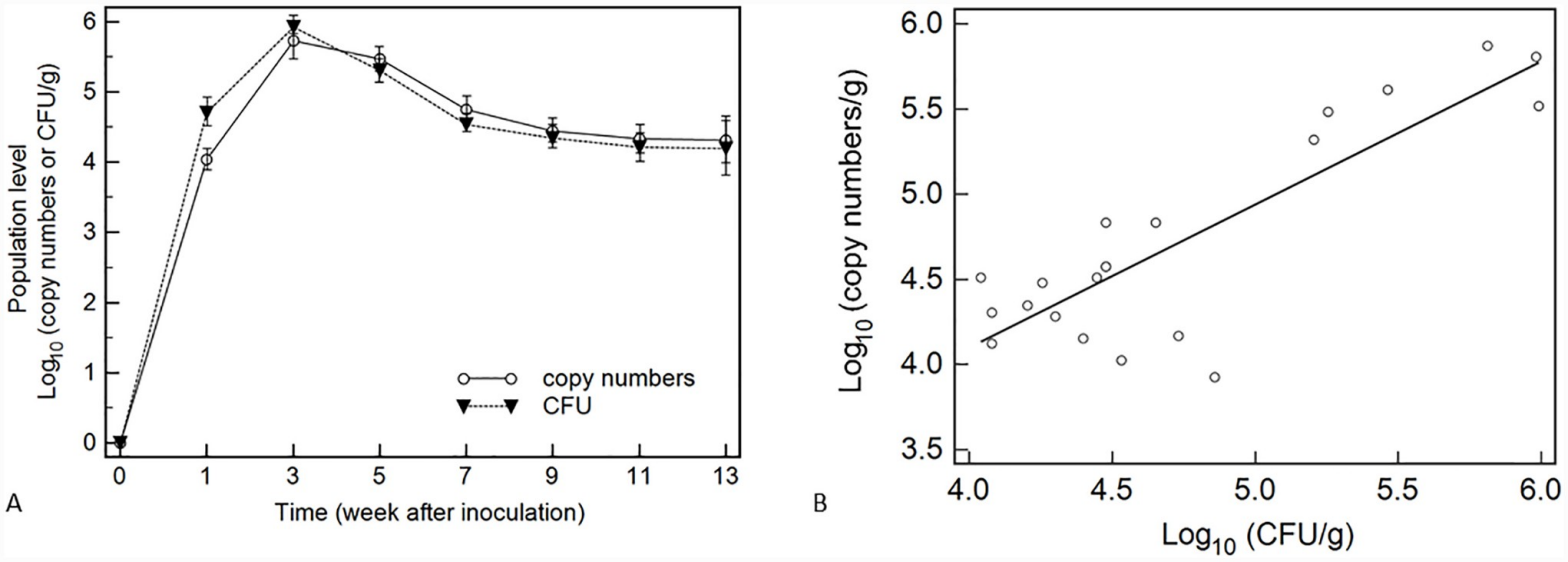
Colonization of transformant G10 in the wheat root-zone soil under greenhouse conditions. A. Population dynamics estimated by real-time PCR and CFU counts. Each point is the average determined from three reaction replicates. B. Correlation between copy number and CFU counts from equal soil samples. The corresponding regressions are y = 0.735 + 0.841x and R^2^ = 0.718. The regressions are significant at *P* < 0.001 (df = 19).

The population dynamics of transformant G10 in root-zone soil are shown in Fig 2A. Log-transformed values estimated with the real-time PCR procedure and by CFU counts at each sampling time present similar population dynamics showing that the trend was consistent. The population of transformant G10 increased quickly over the first 3 weeks, decreased slowly over the following 4 weeks before stabilizing. Specifically, G10 population levels reached 5.4 × 10^5^ copies/g (8.5 × 10^5^ CFU/g) in week 4 following the rapid increase, and then they stabilized in week 13 after the initial decline, reaching 2.1 × 10^4^ copies/g (1.6 × 10^4^ CFU/g). It is noteworthy that no colonies or fluorescence signals were detected in the controls inoculated with wild-type *B*. *bassiana* 08F04 conidial suspension or sterile water. In addition, population levels determined by CFU counts were higher than those of the real-time PCR within the first 3 weeks while they declined faster than the population estimated by the real-time PCR for the following weeks. To comparative analyze the two quantification methods, the results estimated by CFU counts are plotted against the real-time PCR (Fig 2B). A good correlation was obtained in the experiments (y = 0.735 + 0.841x, where y represents the log of the real-time PCR values and x represents the log of the CFU counts; R^2^ = 0.718, *P* < 0.001, df = 19). These results indicate that a detection system using real-time PCR and CFU counts on the selective plates can be used to accurately estimate inoculation strain population densities in a non-sterile environment.

**Fig 3:**
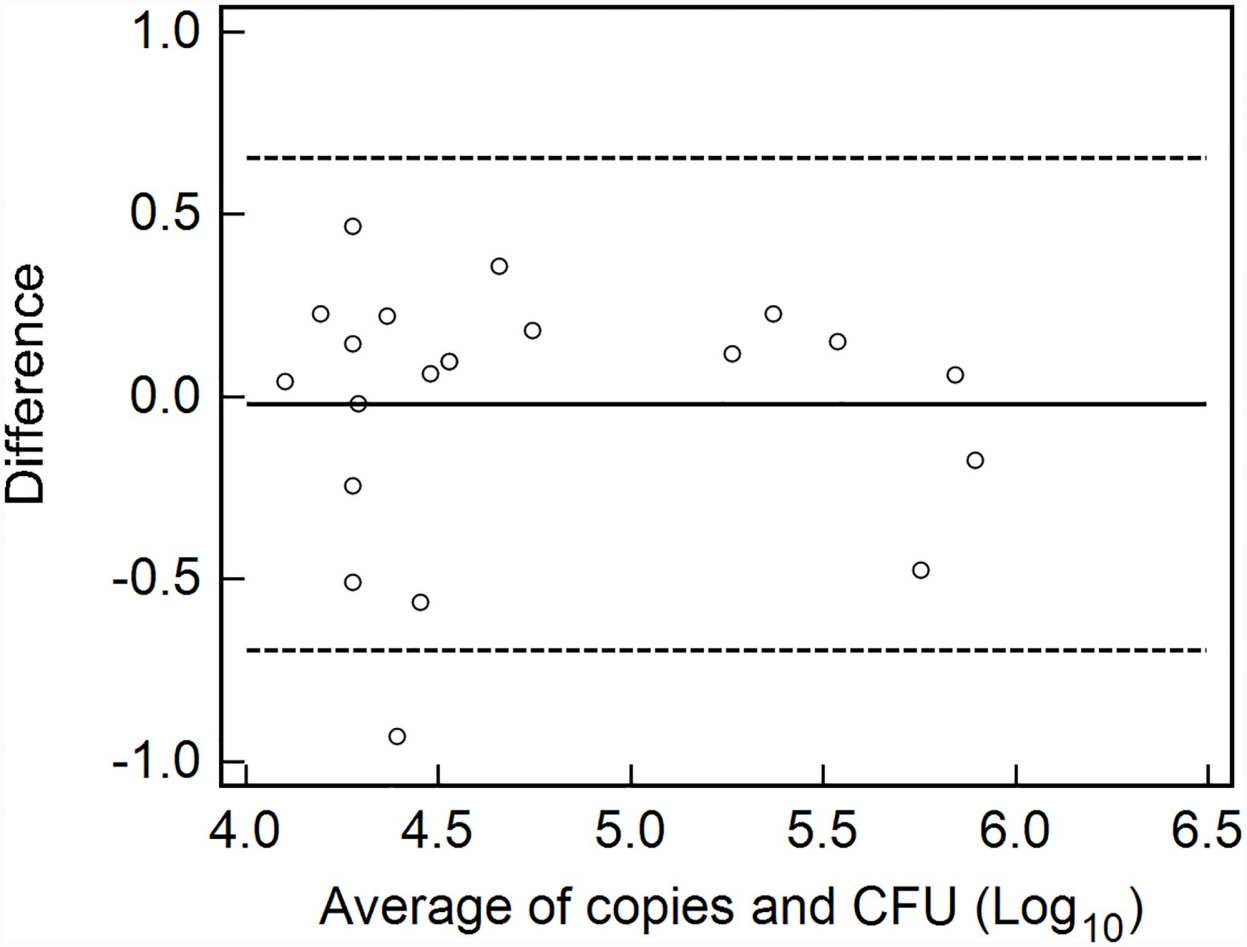
Bland-Altman plot of copy number and CFU counts applied to equal soil samples. Differences between the two methods are plotted against the average of the two methods. The solid line in the center represents the mean of differences. The two dashed lines denote the confidence interval (±1.96 SD). Each point is the average derived from three reaction replicates.

The Bland-Altman test was used to evaluate the agreement of the two methods (Fig 3). The statistical analysis confirms that the results of the two monitoring methods for each sampling time were general in agreement and the mean difference only reached 2.2%. In addition, a divergence appeared in the first sampling time, corresponding to generally low real-time PCR values and higher CFU counts.

### Effect of *Beauveria bassiana* 08F04 and transformant G10 on cereal cyst nematodes in the greenhouse

Analysis of variance showed that there was a significant difference between the three groups (*F*_*2*, *6*_ = 203.1, *P* = 0.0001). Thirteen weeks after planting/inoculation, the application of *B*. *bassiana* 08F04 and transformant G10 had reduced the number of CCN females in the roots significantly compared to the control. Specifically, 64.4% and 60.2% reductions were observed for treatment with *B*. *bassiana* 08F04 and transformant G10, respectively (Table 2). Also, the rate of CCN females parasitized by G10 in soil was 28.5%. No *B*. *bassiana* colonies were isolated from the CCN females in wild-type 08F04 treatment and control on the PDA plate supplemented with hygromycin B (500 μg/mL), streptomycin (100 μg/mL) and kanamycin (50 μg/mL).

**Table 2:** Effects of wild-type *Beauveria bassiana* 08F04 and transformant G10 inoculum on number of females of *Heterodera filipjevi* in wheat roots in the greenhouse after 13 weeks.

Treatment	Females/plant^a^	Proportional reduction (%)
G10	25.3 ± 0.9 b	60.2
08F04	22.7 ± 1.2 b	64.4
Control	63.7 ± 2.4 a	

^a^ Data are mean ± standard error for three replicates. Values followed by the same letter within a column are not significantly different according to ANOVA and LSD test conducted at *P* = 0.05 (*F*_*2*, *6*_ = 203.1, *P* = 0.0001).

## Discussion

Biological control is attracting more attention with mounting pressures to find more sustainable control measures [42]. Research efforts made to screen new antagonists of CCNs have resulted in the isolation of new strains such as *B*. *bassiana* 08F04, which is a promising BCA for controlling CCNs. The colonization capacities of biocontrol fungi in soil are an important factor for the control of soilborne diseases [13]. However, the application of these microbial agents in the field has often failed to achieve the desired results, which is mainly attributable to a lack of knowledge of their colonization in the environment [43]. Thus, it is essential to assess biocontrol fungal colonization and activity in soils. Aggarwal et al. [44] tracked the population of *Chaetomium globosum* in soil using a real-time PCR with specific primers, and their results showed that the initial population of target DNA was 2.5 × 10^8^ copies/g soil increased 10 times 15 days after inoculation. Bonaldi et al. [43] transformed an apramycin resistant gene into *Streptomyces* sp., and the population dynamics of the strain in soil were studied on plates containing apramycin. In this paper, we described an effective means of inserting foreign genes into the *B*. *bassiana* 08F04 genome using ATMT and accomplished with a quantitative determination system using real-time PCR and CFU counts on the selective plates, and the methods employed proven tools for research on the colonization of *B*. *bassiana* in the environment. The availability of these tools will enable further ecological, biological and functional studies of *B*. *bassiana*, and studies of its capacities for the biocontrol of CCN in both semifield and field conditions. Although many researchers have reported on the negative effects of *B*. *bassiana* to insects [45–47], similar studies on plant-parasitic nematodes have been limited. Chen et al. [48] found that *B*. *bassiana* can limit the hatching of *Heterodera glycines*. Liu et al. [49] reported that the culture filtrates of *B*. *bassiana* snf907 strongly inhibit egg hatching and are toxic to J2 of *Meloidogyne hapla*. In this study, the inoculation of conidial suspensions of wild-type *B*. *bassiana* 08F04 and transformant G10 significantly reduced the population densities of CCN females in a greenhouse experiment. The reduction in observed nematode invasion may be due to rhizosphere colonization by *B*. *bassiana* 08F04 as well as decreases of *H*. *filipjevi* J2 density in soil. J2s are the infective stage and penetrate the roots to establish feeding sites, so J2s are a critical stage in the live cycle of CCN [50]. In this study, cultural filtrates of wild-type *B*. *bassiana* 08F04 and transformant G10 showed strongly impacted J2 survival, indicating that their secondary metabolites are likely have nematicidal activity. So the number of CCN females in the roots was reduced following a decline in infective J2s in the soil. In addition, *B*. *bassiana* can produce some extracellular enzymes with nematicidal activity. In our study, we found that the chitinase activity of transformant G37 decreased significantly relative to that of the wild-type strain along with significant increased in *H*. *filipjevi* J2 survival. This phenomenon may be attributed to the fact that a decline in extracellular chitinase reduced the degradation of nematode chitin, which is an important component of nematode cuticles and eggshells [51]. Chitinase is also an important inhibitory factor of *B*. *bassiana* against insects [52]. In addition, AChE is important for postsynaptic transmission in most animals [53]. Cultural filtrates of wild-type *B*. *bassiana* 08F04 and of transformants G10, G37, and G85 decreased AChE activity of *H*. *filipjevi* J2, leading to the functional disturbance of nerves and to lower levels of J2 survival. However, transformant G94 significantly increased AChE activity and J2 survival rates when compared to wild-type *Beauveria* 08F04, and no significant differences were found relative to the control. We also found that the transformation caused a significant change in mycelial and conidial yields of some transformants. As T-DNA was integrated at random chromosomal sites in the host genome and realized insertional mutagenesis [54,55], this procedure can lay the foundation for further investigations of biocontrol mechanisms of *B*. *bassiana* against CCN.

The mode of T-DNA integration observed in the *Agrobacterium* system is determined by the host cell involved [56,57]. In our case, a Southern blot analysis with four transformants was performed and the results indicate that the T-DNA gene was inserted into all of the verified transformant genomes as a single copy and that integration occurred at different chromosomal sites. Fang et al. [58] also reported that most transformants of *B*. *bassiana* contain a single copy and that T-DNA inserts are stably inherited after five generations. Thus, it is speculated that insertions of T-DNA of *B*. *bassiana* into the *Agrobacterium* system are mainly composed of single copies. GFP and its variants have become more commonly used as living reporters in many microorganisms. As the GFP signals can be detected conveniently and obviously without manipulating samples, the application of GFP technology has become the most promising approach in the era of functional genomics [59,60]. Thus, the protocol described here can facilitate investigations into the molecular mechanisms of *B*. *bassiana* 08F04.

Transformant G10 was used for our pilot study of the colonization and population dynamics of *B*. *bassiana* in the environment, as it is similar to the wild-type strain in terms of growth and biocontrol, and can accurately reflect colonization of *B*. *bassiana* 08F04. In addition, a non-sterile soil was used to stimulate the introduction of the strain to a resident population of microorganisms and to thereby assess the competitiveness of *B*. *bassiana* 08F04. The introduced population of G10 was found to survive in soil, although a decline in its density was observed over 3 weeks after bulk soil inoculation. Population dynamics of the introduced microbial population are attributed to a scarcity of available nutrients and adverse factors including biotic and abiotic aspects [61,62]. However, after an initial decline in population, the G10 population remained stable for 13 weeks, which may indicate the establishment of an equilibrium with the resident microorganisms. The population dynamics of G10 were found to be similar with those of the other BCAs described previously [42, 63–65]. Therefore, *B*. *bassiana* 08F04 can stably colonize soil and effectively reduce the population of CCN females 13 weeks after inoculation. To achieve the best control, the supplementary use of BCA or of other control methods could be applied 3 weeks after inoculation.

The population level indicated by the CFU counts was higher than that determined by real-time PCR over the first 3 weeks, however, it declined faster over the following weeks relative to the real-time PCR results. This is likely to be due to the residue of non-degraded DNA in the soil detected by the real-time PCR. DNA degradation rates of nucleases after cell death are strongly depend on environmental conditions [66–68]. The results of the present study indicate that DNA from dead cells can persist for a certain period under greenhouse conditions.

The present study showed the combined use of real-time PCR and CFU counts is useful for tracking the population of an inoculum strain under non-sterile soil conditions and can generate valuable information on population behavior, facilitating the rational use of this BCA in the field. Further studies must perfect and simplify these methods not only to determine the influence of *B*. *bassiana* 08F04 colonization on the diversity of resident soil microorganisms but also to facilitate research on the interaction mechanisms of *B*. *bassiana* and CCN.

## Conclusions

In the current study, successful procedures for *B*. *bassiana* 08F04 genetic transformation by ATMT and for the quantification of target genes by real-time PCR were explored. The transformations obtained caused significant changes in growth rate and biocontrol capacities for some transformants. Transformant G10 was used in a pilot colonization study of *B*. *bassiana*, as it was similar with the wild-type strain in terms of growth rate and biocontrol capacities. The results obtained by real-time PCR and CFU counts indicated similar population dynamics and indicated that both the two methods was available and they should be chosen according to the purposes of future research. However, for other target organisms, direct culture from soil might not be feasible, so using genetically marked strains might be the only option. The population of G10 were enhanced within the first 3 weeks, declined slowly over the following 4 weeks before stabilizing. The application of wild-type *B*. *bassiana* 08F04 and transformant G10 also significantly reduced the population of CCN females in roots. The results of this study can help guide ecological, biological and functional studies of *B*. *bassiana* 08F04, as well as bionematicide registration.

## Supporting information

S1 Fig Real-time PCR standard curve determined from dilution series of pCAM-gfp plasmids. The corresponding regressions apply the following: y = 39.9–3.08x, R^2^ = 0.994, and E = 111%.
(TIF)

S1 Table Growth and sporulation of *Beauveria bassiana* 08F04 transformants on potato dextrose agar. (DOCX)

S2 Table Extracellular enzyme activity of four transformants and wild-type *Beauveria bassiana* 08F04. (DOCX)

S1 Raw Images (PDF)
