# Supplementary figures and images for: Colonization of Beauveria bassiana 08F04 in root-zone soil and its biocontrol of cereal cyst nematode (Heterodera filipjevi)
Source: PLoS One. 2020 May 5;15(5):e0232770. doi: 10.1371/journal.pone.0232770 (PMC7199937; doi:10.1371/journal.pone.0232770)

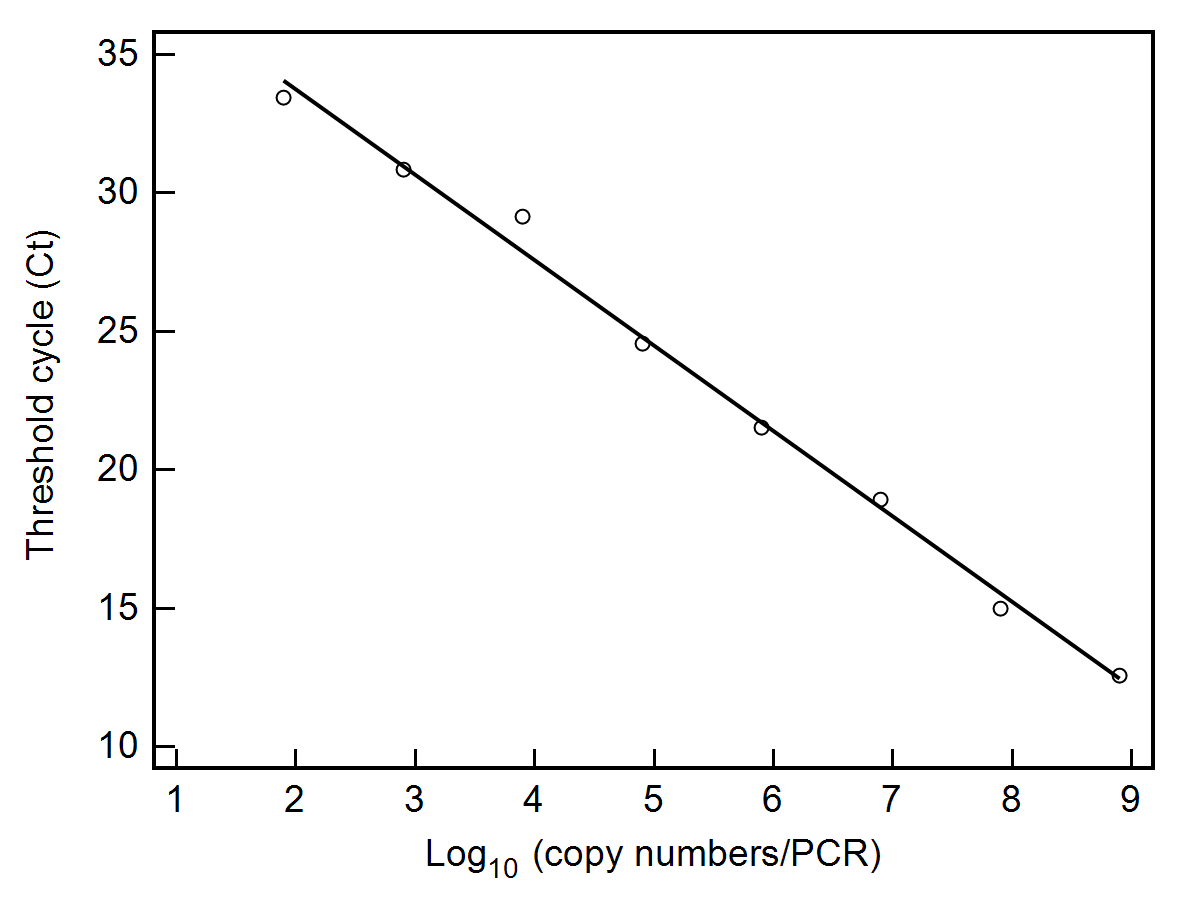

Supplement: S1 Fig — The corresponding regressions apply the following: y = 39.9–3.08x, R2 = 0.994, and E = 111%. (TIF) [file pone.0232770.s001.tif]
