# Supplementary material for: Colonization of Beauveria bassiana 08F04 in root-zone soil and its biocontrol of cereal cyst nematode (Heterodera filipjevi)
Source: PLoS One. 2020 May 5;15(5):e0232770. doi: 10.1371/journal.pone.0232770 (PMC7199937; doi:10.1371/journal.pone.0232770)
Supplement: S1 Table — (DOCX) [file pone.0232770.s002.docx]

**S1 Table Growth and sporulation of *Beauveria bassiana* 08F04 transformants on potato dextrose agar**

| **Strain** | **Mycelial yield (g/L)^a^** | **Conidial yield (10^9^ conidia/mL)^b^** |
| --- | --- | --- |
| G6 | 5.61 ± 0.15 d | 2.67 ± 0.12 ab |
| G10 | 6.50 ± 0.12 bc | 2.10 ± 0.12 cd |
| G21 | 3.63 ± 0.27 e | 2.97 ± 0.23 a |
| G37 | 6.97 ± 0.12 b | 1.70 ± 0.06 de |
| G59 | 7.83 ± 0.22 a | 0.97 ± 0.15 f |
| G67 | 6.67 ± 0.09 bc | 2.60 ± 0.12 ab |
| G69 | 8.07 ± 0.20 a | 1.60 ± 0.06 e |
| G85 | 6.27 ± 0.20 c | 2.27 ± 0.09 bc |
| G94 | 6.37 ± 0.12 c | 1.93 ± 0.09 cde |
| 08F04 | 6.53 ± 0.09 bc | 2.13 ± 0.22 cd |

^a^ Data are mean ± standard error for three replicates; Values followed by the same letter shown in columns are not significantly different according to ANOVA and LSD test conducted at *P* = 0.05; *F* _9, 20_ = 56.8, *P* = 2.8×10^-12^. ^b^ *F*_9, 20_ = 18.3, *P* = 8.5×10^-8^.
