# Supplementary material for: Colonization of Beauveria bassiana 08F04 in root-zone soil and its biocontrol of cereal cyst nematode (Heterodera filipjevi)
Source: PLoS One. 2020 May 5;15(5):e0232770. doi: 10.1371/journal.pone.0232770 (PMC7199937; doi:10.1371/journal.pone.0232770)
Supplement: S2 Table — (DOCX) [file pone.0232770.s003.docx]

**S2 Table Extracellular enzyme activity of four transformants and wild-type *Beauveria bassiana* 08F04**

| Strain | Protease (U/mL)^a^ | Chitinase (U/mL)^b^ |
| --- | --- | --- |
| G10 | 41.0 ± 0.8 ab | 21.2 ± 1.4 a |
| G37 | 42.5 ± 2.4 a | 10.9 ± 2.3 b |
| G85 | 39.4 ± 0.5 ab | 23.0 ± 2.3 a |
| G94 | 37.2 ± 0.5 b | 21.7 ± 1.7 a |
| 08F04 | 40.4 ± 0.7 ab | 21.5 ± 1.4 a |

^a^ Data are mean ± standard error of three replicates; Values followed by the same letter shown in columns are not significantly different according to ANOVA and LSD test conducted at *P* = 0.05; *F_4, 10_* = 2.8, *P* = 0.086. ^b^ *F_4, 10_* = 7.1, *P* = 0.006.
