# Supplementary material for: Colonization of Beauveria bassiana 08F04 in root-zone soil and its biocontrol of cereal cyst nematode (Heterodera filipjevi)
Source: PLoS One. 2020 May 5;15(5):e0232770. doi: 10.1371/journal.pone.0232770 (PMC7199937; doi:10.1371/journal.pone.0232770)
Supplement: S1 Raw Images — (PDF) [file pone.0232770.s004.pdf]

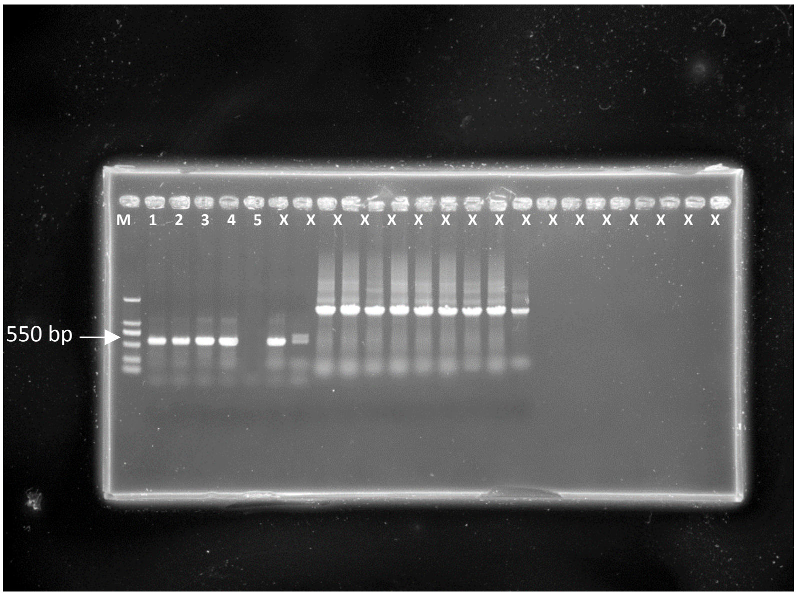

Fig 1D. PCR products of the *sgfp* gene of the genomic DNA of the transformants (lanes 1-4: transformants G10, G37, G85 and G94) and of wild-type *B. bassiana* 08F04 (lane 5)

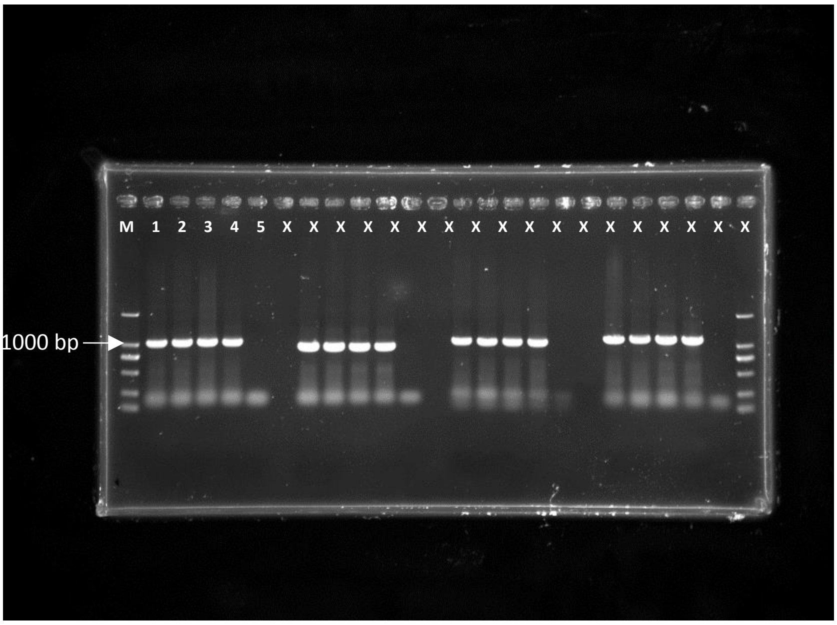

Fig 1E. PCR products of the hph gene of the genomic DNA of the transformants (lanes 1-4: transformants G10, G37, G85 and G94) and of wild-type *B. bassiana* 08F04 (lane 5)

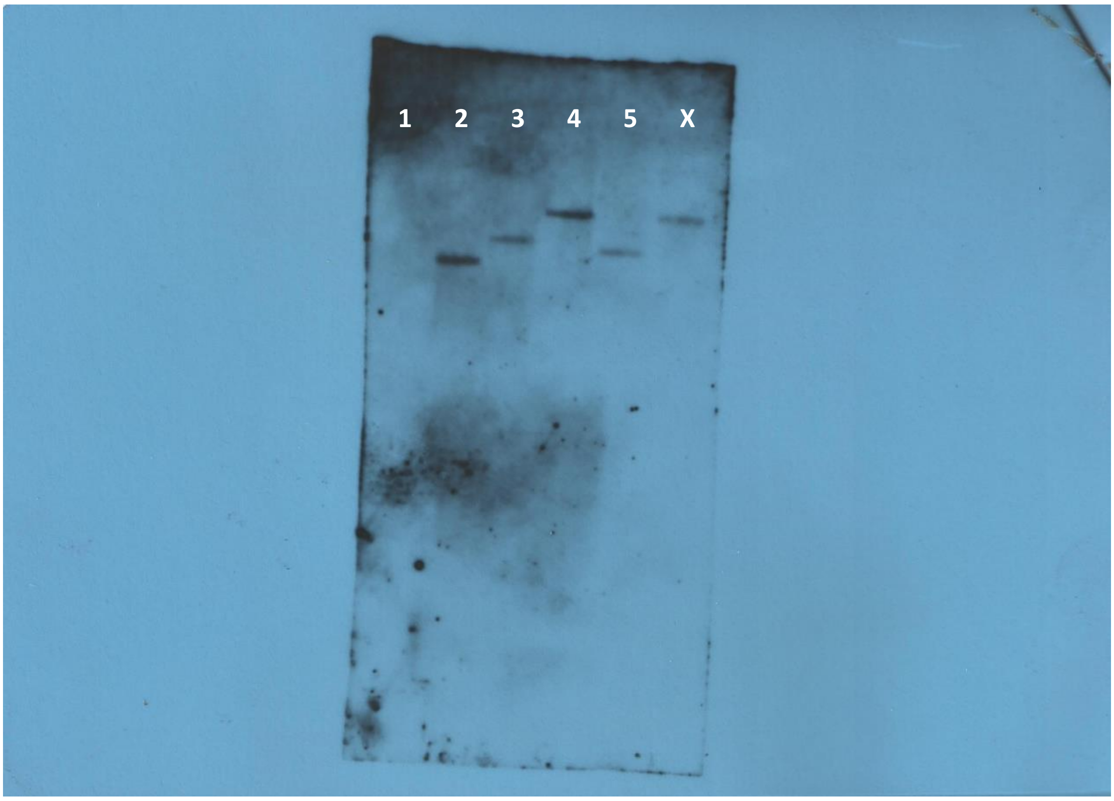

Fig 1F. Southern blot analysis with a DIG-labeled *sgfp* gene probe of wild-type 08F04 (lane1) and of the transformants (lanes 2-5: transformants G10, G37, G85 and G94).
